# Brain structural MRI marker for predicting conversion to Parkinson’s disease in individuals with prodromal symptoms

**DOI:** 10.3389/fnagi.2025.1579326

**Published:** 2025-07-16

**Authors:** Chang-hyun Park, Uicheul Yoon, Phil Hyu Lee, Jinna Kim, Seung-Koo Lee, Na-Young Shin

**Affiliations:** ^1^Division of Artificial Intelligence and Software, College of Artificial Intelligence, Ewha Womans University, Seoul, Republic of Korea; ^2^Department of Biomedical Engineering, College of Bio and Medical Sciences, Daegu Catholic University, Gyeongsan, Republic of Korea; ^3^Department of Neurology, Yonsei University College of Medicine, Seoul, Republic of Korea; ^4^Department of Radiology, Research Institute of Radiological Science, and Center for Clinical Imaging Data Science, Yonsei University College of Medicine, Seoul, Republic of Korea

**Keywords:** Parkinson’s disease, prodromal symptom, olfactory dysfunction, rapid eye movement sleep behavior disorder, MRI, machine learning

## Abstract

**Background:**

During the prodromal stage of Parkinson’s disease (PD), brain structural alterations precede clinical diagnosis and offer opportunities for early detection. We investigated whether combining clinical non-motor markers with an MRI-based brain structural marker could enhance predictive performance for PD conversion.

**Methods:**

Individuals with prodromal symptoms (*n* = 46, 63.5 ± 7.6 years, 24 males) were selected from the Parkinson’s Progression Markers Initiative dataset. We developed a machine learning classifier to identify individuals with brain structural patterns similar to PD based on cortical thickness and white matter integrity. Its predictive performance for PD conversion was assessed alone and combined with clinical non-motor markers such as rapid eye movement sleep behavior disorder and olfactory dysfunction.

**Results:**

Six individuals converted to PD within 4 years. The MRI marker classified 21 individuals as having PD-like brain patterns, including all six converters. When combined with olfactory dysfunction, the approach achieved optimal performance with 100% sensitivity, 80% specificity, and 90% balanced accuracy, outperforming individual markers and other combinations.

**Conclusion:**

MRI-quantified brain structural similarity to PD, particularly when combined with olfactory assessment, significantly enhances prediction of PD conversion in individuals with prodromal symptoms. This accessible, multimodal approach could facilitate early identification of high-risk individuals for targeted interventions and clinical trials.

## 1 Introduction

The prodromal stage of Parkinson’s disease (PD), termed prodromal PD, is a phase where neurodegenerative pathology initiates but motor symptoms necessary for PD diagnosis are not yet present. Prodromal PD has been receiving increasing attention since disease-modifying treatments for delaying or even stopping the progression of the disease could be suitably tested for it (Mahlknecht et al., 2022). However, identifying prodromal PD and predicting conversion to PD among individuals with non-specific prodromal features remain challenges, despite progress in understanding its pathophysiology and clinical manifestations.

During the years to decades of prodromal PD (Schenck et al., 2013), nigral neurodegeneration and extranigral Lewy body pathology (Del Tredici and Braak, 2012) could manifest as non-specific clinical signs and symptoms that serve as prodromal markers. Given that the initial PD pathology often originates outside the substantia nigra, particularly in the lower brainstem or olfactory bulb (Braak et al., 2003; Beach et al., 2009), these neuropathological changes increase the probability of developing specific non-motor phenomena such as rapid eye movement sleep behavior disorder (RBD) and olfactory impairment (hyposmia or anosmia), presenting a significant opportunity for identifying prodromal PD through targeted screening (Khoo et al., 2013). The occurrence of RBD or olfactory deficits not only signifies potential underlying neurodegeneration but also suggests an elevated risk for the emergence of additional prodromal features (Gaenslen et al., 2014).

While the presence of specific markers does not invariably lead to conversion from prodromal PD to PD, the ability to predict conversion to PD has been supported by the strongest evidence for both RBD and olfactory dysfunction (Postuma and Berg, 2016) but with varying degrees of sensitivity and specificity. The low prevalence of RBD in the general population, coupled with its high long-term risk of developing PD (Iranzo et al., 2014), accentuate its specificity over sensitivity; conversely, the high prevalence of olfactory dysfunction in PD as well as in the general population (Doty, 2007) underlines its sensitivity over specificity [for a summary, see (Postuma et al., 2012a)]. We hypothesized that such limitations in the predictive abilities of clinical non-motor markers could be compensated by combining them with neuroimaging markers that provide complementary information about underlying pathophysiological processes. Specifically, we expected that imaging-based markers reflecting early neurodegeneration could optimize the balance between sensitivity and specificity when combined with clinical assessments that have complementary predictive profiles. In this regard, not only did we attempt a combination of the dopamine transporter (DaT) scan marker, which is well-known for its high sensitivity and specificity (Ba and Martin, 2015), with clinical non-motor markers, but we also proposed a new imaging marker derived from MRI and evaluated its combination with clinical non-motor markers for predicting conversion to PD. Considering that progressive neurodegeneration could induce brain structural alterations even before the clinical diagnosis of PD (Yang et al., 2021; Pimer et al., 2023), individuals whose brain structural patterns are closer to PD brains would be likely to be at higher risk of developing PD than those whose brain structural patterns are closer to healthy brains. Therefore, we aimed to develop an MRI-based classifier using a machine learning method to discriminate PD brains from healthy ones, then applied it to individuals with prodromal symptoms to assess its performance for predicting PD conversion.

## 2 Materials and methods

### 2.1 Study sample

**FIGURE 1:**
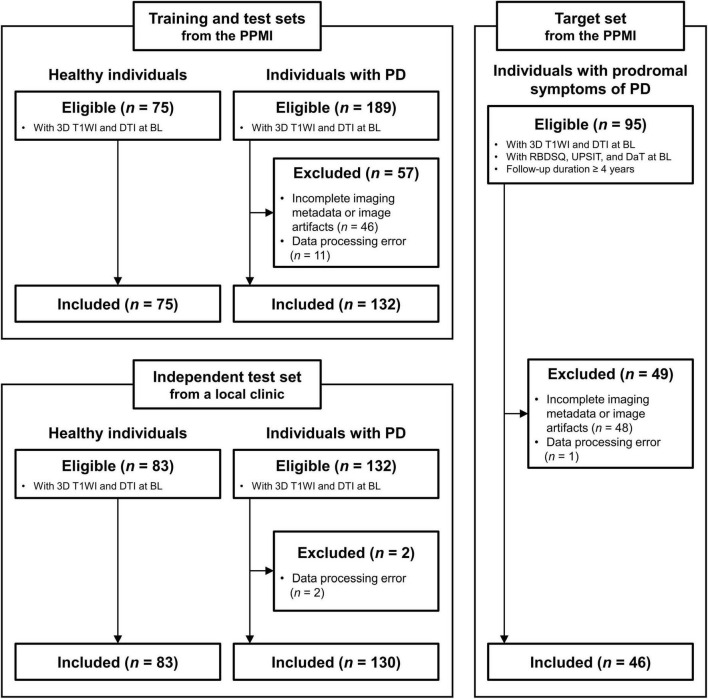
Flowchart depicting the inclusion/exclusion process for study population. BL, baseline; DaT, dopamine transporter imaging; DTI, diffusion tensor imaging; PD, Parkinson’s disease; PPMI, Parkinson’s Progression Markers Initiative; RBDSQ, rapid eye movement sleep behavior disorder screening questionnaire; T1WI, T1-weighted imaging; UPSIT, University of Pennsylvania smell identification test.

A total of 46 individuals with prodromal symptoms of PD (mean ± standard deviation age: 63.5 ± 7.6 years, 24 males) who were followed up for more than 4 years were included from the Parkinson’s Progression Markers Initiative (PPMI) dataset.^1^ For developing an MRI-based classifier, we included 75 healthy individuals (mean ± standard deviation age: 59.9 ± 11.3 years, 49 males) and 132 individuals with PD (mean ± standard deviation age: 60.7 ± 9.2 years, 88 males) from the PPMI dataset to serve as training and test sets. An independent test set of 83 healthy individuals (mean ± standard deviation age: 66.4 ± 8.2 years, 50 males) and 130 individuals with PD (mean ± standard deviation age: 69.0 ± 9.6 years, 72 males) was incorporated from a local tertiary hospital dataset (Figure 1). More details on inclusion and exclusion criteria are described in Supplementary Appendix S1. This study was approved by the institutional review board, and a waiver for written consent was obtained because of the retrospective study design.

### 2.2 MRI data collection and preprocessing

Locally recruited participants underwent 3T MRI scans (Philips Healthcare, Best, Netherlands), while PPMI dataset participants’ scans were acquired using site-specific MRI systems from various manufacturers. Acquisition parameters for structural T1-weighted and diffusion-weighted MRI data according to the datasets are summarized in Supplementary Table S1.

To mitigate potential scanner-related effects, all MRI data underwent standardized preprocessing pipelines with consistent parameters across all datasets. For structural T1-weighted images, preprocessing included skull stripping, intensity scaling, bias field correction, and spatial normalization using the CIVET pipeline^2^ for cortical thickness extraction. For diffusion-weighted images, preprocessing steps included denoising, Gibbs ringing removal, motion and eddy current correction, intensity scaling, bias field correction, and spatial normalization using FSL tools,^3^ followed by tract-based spatial statistics (TBSS) analysis for fractional anisotropy extraction.

### 2.3 Brain structural features

Since cortical thickness in gray matter (GM) regions and integrity in white matter (WM) regions could adequately serve as features to infer the progression of brain structural alterations in PD (Park et al., 2022), we adopted these features (cortical thickness from 62 GM regions and integrity from 48 WM regions) to develop the classifier to distinguish between healthy and PD brains (Supplementary Appendix S2). All 62 cortical thickness and 48 white matter integrity measures were used without feature selection to preserve model interpretability and examine individual contributions of the complete feature set, while avoiding potential overfitting that can occur with feature selection in small datasets.

### 2.4 Classifier to distinguish between healthy and PD brains

The MRI-based classifier was generated by employing the random forest method that works by training an ensemble of decision trees on random subsets of a training set and making predictions by summarizing the predictions of all the decision trees. By employing the brain structural features along with the confounding variables of age and sex as predictors, the classifier was trained on a training set and subsequently evaluated for performance on a test set, as well as a separate, independent test set, in distinguishing between healthy and PD brains (Supplementary Appendix S3). The independent test set was introduced to assess generalizability across different scanner specifications and acquisition protocols, allowing evaluation of our approach’s robustness to inter-scanner variability without requiring explicit harmonization techniques. The overall performance of the classifier was assessed by measuring the accuracy of predictions and the area under a receiver operating characteristic curve (AUC).

### 2.5 Division of prodromal individuals into close-to-healthy and close-to-PD

The MRI-based classifier was applied to the prodromal individuals as a target set to classify them. When the prodromal individuals were classified as PD by this model, their brain structural patterns were considered to more closely resemble those of the PD individuals rather than the healthy individuals. We designate this as the “close-to-PD” state, indicating that from a brain structural pattern perspective, these individuals appear neuroanatomically closer to PD than to healthy conditions. Similarly, the prodromal individuals classified as healthy were designated as being in the “close-to-healthy” state. We emphasize that these designations are exploratory classifications proposed in this study based on brain structural patterns, rather than established clinical categories from the literature. Given the random forest model, the PD class probability, that is, the probability of each individual belonging to the PD class was computed as the proportion of trees in the forest that predicted the PD class of the individual.

Clinical characteristics of the prodromal individuals according to their designated states were assessed relative to the healthy and PD individuals by comparing their scores on a set of clinical assessments, including the Hoehn and Yahr staging scale (HYSS), Movement Disorders Society-sponsored revision of the unified PD rating scale part III (MDS-UPDRS III), RBD screening questionnaire (RBDSQ), and University of Pennsylvania smell identification test (UPSIT), using a confounder-adjusted permutation test (Supplementary Appendix S4). Statistical significance was determined at a *p*-value < 0.05, corrected for *post hoc* multiple comparisons using a false discovery rate method.

### 2.6 Brain structural contributions to the division of prodromal individuals

It is important to note that our approach to predicting PD conversion differs from conventional prediction models. Rather than directly training a model on prodromal individuals to predict PD conversion, we employed the healthy-PD classification model as a proxy. This methodological choice was primarily based on our fundamental assumption that neurodegeneration-related brain structural changes precede clinical PD diagnosis, making individuals with brain patterns resembling PD more likely to develop the disease than those with healthy-like patterns. The practical constraint of limited available prodromal individuals and confirmed PD conversion cases in existing datasets further supported this approach. Consequently, our sensitivity analysis for determining how input variables affected model outputs in PD conversion prediction relied on analyzing the underlying healthy-PD classification model. As sensitivity analysis, to identify which brain regions were important to discriminate the PD and close-to-PD states from the healthy and close-to healthy states, respectively, we evaluated the consistency and robustness of the classifier’s feature importances across different data samples (test and target sets) using Shapley additive explanations (SHAP) analysis (Supplementary Appendix S5). The overall importance of each feature was assessed by SHAP magnitude, calculated as the mean of absolute SHAP values across samples in the respective set.

### 2.7 Development of PD in prodromal individuals

For the prodromal individuals, PD conversion was determined by confirming their clinical diagnosis beyond 4 years, such that those diagnosed with idiopathic PD during follow-up at or before 4 years, with unchanged diagnosis thereafter, were regarded as incident PD cases.

### 2.8 Performance of PD conversion predictors

To assess an increased risk of developing PD, we considered the following four markers: (i) RBD assessed using the RBDSQ [RBD], defined as RBD positive with a score of 5 or higher on the questionnaire evaluating sleep behavior, (ii) olfactory dysfunction identified through the UPSIT [Hyposmia], classified as hyposmia or anosmia according to age- and sex-adjusted criteria (males scoring 33 or lower and females scoring 34 or lower), (iii) close-to-PD determined by the MRI-based classifier [MRI], and (iv) nigrostriatal dopaminergic degeneration assessed through visual analysis of DaT scans [DaT]. Detailed evaluation methods for the markers are provided in Supplementary Appendix S6.

We evaluated a total of 11 predictors for conversion to PD by examining the scenarios when only clinical non-motor markers were available, as well as when both clinical non-motor and imaging markers were available. Specifically, predictors comprising only clinical non-motor markers included [RBD] and [Hyposmia] individually and their concomitance [RBD + Hyposmia]. Additionally, we considered predictors not only composed of [MRI] and [DaT] individually but also their concurrent presence with clinical non-motor markers (for [MRI], [MRI] alone, [MRI + RBD], [MRI + Hyposmia], [MRI + RBD + Hyposmia]; and similarly, for [DaT], [DaT] alone, [DaT + RBD], [DaT + Hyposmia], [DaT + RBD + Hyposmia]). The integration of multiple markers was systematically implemented through assessment of their concurrent presence in each individual. For example, for the integration of the MRI-based classifier ([MRI]) with clinical non-motor markers ([RBD] or [Hyposmia]), our methodology specifically examined whether both markers occurred simultaneously in the same individual. For each individual, the presence of multiple markers was recorded as a binary outcome (present/absent) based on the simultaneous occurrence of all component markers.

The performance of each predictor for PD conversion was evaluated firstly by conducting Fisher’s exact test under the null hypothesis of no non-random association between predicted and actual PD conversion outcomes, and secondly by computing performance metrics quantifying the concordance between the predicted and observed instances of PD conversion, including sensitivity, specificity, positive predictive value (PPV), and negative predictive value (NPV), and for a comprehensive assessment of performance, balanced accuracy (BA) and Matthews correlation coefficient (MCC) (Supplementary Appendix S7).

### 2.9 Software and code availability

In this study, the development of the MRI-based classifier and SHAP analysis were conducted using respective Python packages. Statistical inferences using the confounder-adjusted permutation test were performed using a MATLAB-based in-house program. The generated MRI-based classifier and the code for the confounder-adjusted permutation test are publicly available at a GitLab repository.^4^

## 3 Results

### 3.1 Clinical characteristics of study population

Table 1 summarizes demographic and clinical characteristics of the healthy individuals (*n* = 75), prodromal PD individuals (*n* = 46), and PD individuals (*n* = 132) from the PPMI dataset.

**TABLE 1:** Demographic and clinical characteristics of individuals included in the study.

Characteristic	Healthy (*n* = 75)	Prodromal (*n* = 46)	PD (*n* = 132)	*P-*value*	*Post hoc* comparison
Age (y)	61.3 (53.1–67.5)	63.7 (60.5–67.8)	62.3 (54.3–67.7)	0.213	
Sex^†^				0.199	
Male	49 (65)	24 (52)	88 (67)		
Female	26 (35)	22 (48)	44 (33)		
HYSS	0 (0–0)	0 (0–0)	2 (1–2)	<0.001	Healthy < PD Prodromal < PD
MDS-UPDRS III	0 (0–1)	2 (0–3)	19.5 (13–25.5)	<0.001	Healthy < PD Healthy < Prodromal Prodromal < PD
RBDSQ	3 (1–4)	4 (2–7)	3 (2–5)	0.002	Healthy < PD Healthy < Prodromal
RBD^†^	15 (20)	9 (20)	45 (34)		
UPSIT	35 (32–37)	32 (19–35)	23 (16–30)	<0.001	Healthy < PD Healthy < Prodromal Prodromal < PD
Olfactory dysfunction^†^	35 (47)	23 (50)	111 (84)		

Values are presented as medians with interquartile ranges in parentheses, unless otherwise noted. HYSS, Hoehn and Yahr staging scale; PD, Parkinson’s disease; RBD, rapid eye movement sleep behavior disorder; RBDSQ, rapid eye movement sleep behavior disorder screening questionnaire; MDS-UPDRS III, Movement Disorder Society-sponsored revision of the unified PD rating scale part III; UPSIT, University of Pennsylvania smell identification test. *P-values were calculated using different statistical methods depending on the nature of the variables: the χ^2^ test was employed for the categorical variable such as sex, the Kruskal-Wallis test was used for the continuous variable like age, and a confounder-adjusted permutation test that controlled for age and sex was applied for other continuous variables.

^†^Data are presented as counts of individuals, with corresponding percentages shown in parentheses.

### 3.2 Classifier to distinguish between healthy and PD brains

The performance of the MRI-based classifier, generated using the brain structural features from 62 GM regions measuring cortical thickness and 48 WM regions assessing integrity, exhibited an accuracy of 0.927 and an AUC of 0.982 on the test set. This classifier based on both types of features outperformed that based solely on features of either cortical thickness (accuracy = 0.805, AUC = 0.923) or WM integrity (accuracy = 0.854, AUC = 0.933). In external validation with the independent test set, the classifier using both types of features (accuracy = 0.873, AUC = 0.949) also outperformed that based solely on either cortical thickness (accuracy = 0.657, AUC = 0.770) or WM integrity (accuracy = 0.864, AUC = 0.885).

### 3.3 Division of prodromal individuals into close-to-healthy and close-to-PD

**FIGURE 2:**
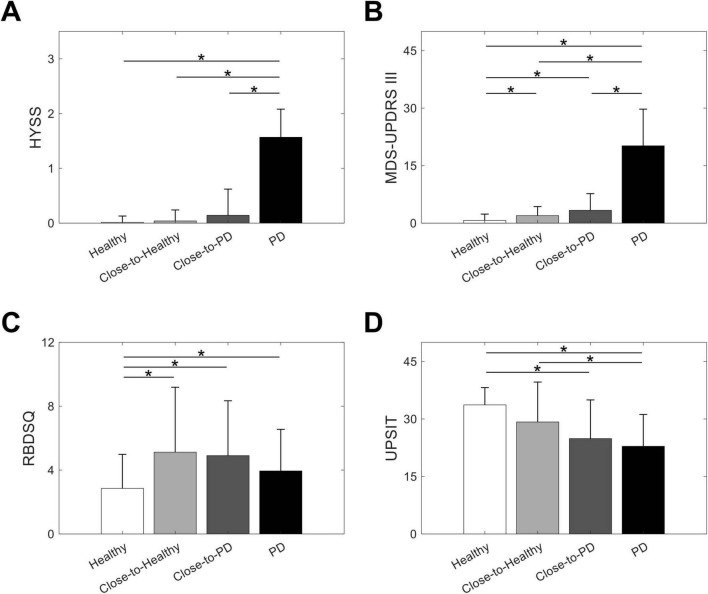
Comparison of scores on clinical assessments. Clinical characteristics of individuals with prodromal symptoms of Parkinson’s disease (PD) in the close-to-healthy or close-to-PD state are assessed relative to healthy individuals and those with PD by comparing scores on the **(A)** Hoehn and Yahr staging scale (HYSS), **(B)** Movement Disorder Society-sponsored revision of the unified PD rating scale part III (MDS-UPDRS III), **(C)** rapid eye movement sleep behavior disorder screening questionnaire (RBDSQ), and **(D)** University of Pennsylvania smell identification test (UPSIT). The bars and error bars represent the mean and standard deviation, respectively, of scores. Statistically significant differences in *post hoc* pair-wise comparisons are indicated by asterisks above the compared groups.

When the MRI-based classifier was applied to the prodromal individuals, 21 were assigned to the close-to-PD state and the other 25 were assigned to the close-to-healthy state. PD class probabilities were significantly higher for prodromal individuals in the close-to-PD state compared to those in the close-to-healthy state. While close-to-healthy prodromal individuals showed similar probabilities to healthy individuals, close-to-PD prodromal individuals had lower probabilities than PD individuals (Supplementary Figure S1 and Supplementary Appendix S8). Scores on clinical assessments revealed that the prodromal individuals in either state displayed clinical characteristics that were intermediate between the healthy and PD individuals. For any clinical assessment, however, there was no significant difference in scores between the prodromal individuals in the close-to-healthy and close-to-PD states (Figure 2 and Supplementary Appendix S9).

### 3.4 Brain structural contributions to the division of prodromal individuals

**FIGURE 3:**
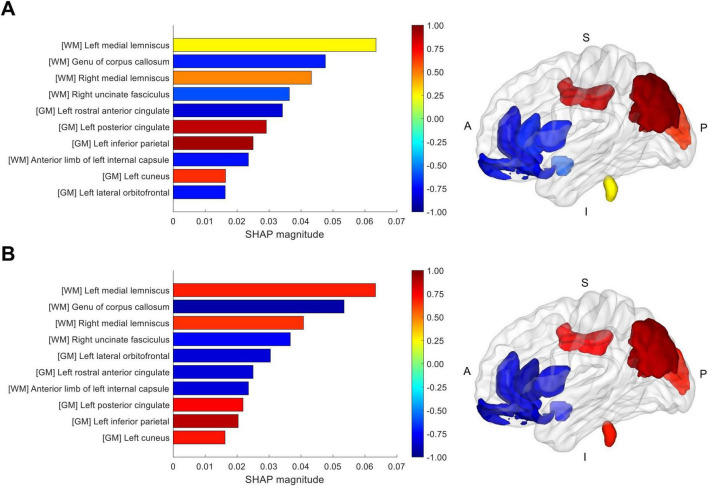
Magnitude of Shapley additive explanations (SHAP) for the top 10 brain structural features. SHAP magnitude, calculated as the mean of absolute SHAP values across samples, reflects the features’ contributions to the classifier’s prediction for **(A)** dividing prodromal individuals into the close-to-healthy and close-to-PD states and for **(B)** distinguishing between healthy and PD brains. The bar plot on the left displays the features ranked by SHAP magnitude, while the rendered brain on the right illustrates the corresponding regions associated with these features. In both plots, the bidirectional color scale of cool and warm hues denotes the directionality of correlation between feature values and SHAP values across samples. Cool colors (negative correlation) indicate that decreasing feature values drive the prediction toward the close-to-PD or PD state, whereas warm colors (positive correlation) signify the opposite trend. WM, white matter; GM, gray matter; S, superior; I, inferior; A, anterior; P, posterior.

The SHAP analysis revealed consistent and robust contributions of brain regions to classifier predictions, with white matter integrity features demonstrating relatively greater impact compared to cortical thickness measures. The correlation between SHAP magnitudes derived from distinguishing healthy and PD brains and those from dividing prodromal individuals into close-to-healthy and close-to-PD states was highly significant (*r* = 0.980, *p* < 0.001), demonstrating consistent feature importance across different classification tasks. Specifically, regions showing the highest predictive importance included several white matter regions such as the left medial lemniscus, genu of corpus callosum, right medial lemniscus, and right uncinate fasciculus, along with cortical regions including the left rostral anterior cingulate, left posterior cingulate, and left inferior parietal cortices. These findings are consistent with established patterns of brain structural changes in PD, including the anterior-to-posterior progression of pathology (Potgieser et al., 2014) and the temporal precedence of white matter over cortical alterations (Park et al., 2022; Figure 3, Supplementary Figure S2, and Supplementary Appendix S10).

### 3.5 Development of PD in prodromal individuals

Among the 21 prodromal individuals classified as the close-to-PD state, six developed PD within 4 years of follow-up. None of the 25 prodromal individuals classified as the close-to-healthy state developed PD during the follow-up.

### 3.6 Performance of predictors for PD conversion within four-year follow-up

The presence of [Hyposmia], [MRI], and [DaT], as well as their combinations of [MRI + Hyposmia] and [DaT + Hyposmia], at baseline was associated with PD conversion within 4 years of follow-up, as demonstrated by Fisher’s exact test, which showed significantly higher odds of developing PD in individuals exhibiting these predictors compared to those who did not (Supplementary Table S2).

Among clinical non-motor markers, as consistently demonstrated in prior studies (Postuma et al., 2012a; Mahlknecht et al., 2015), [RBD] demonstrated relatively high specificity, whereas [Hyposmia] exhibited relatively high sensitivity. The combination of [RBD + Hyposmia] resulted in improved specificity, but it did not maintain the high sensitivity associated with [Hyposmia] alone. While both imaging markers surpassed [RBD] in sensitivity and exceeded [Hyposmia] in specificity, [DaT] showed a balanced profile of high sensitivity and specificity, and [MRI] displayed lower specificity but higher sensitivity compared to [DaT]. The combination of [DaT + RBD] and [DaT + Hyposmia] enhanced specificity relative to using individual clinical non-motor markers alone, but it diminished sensitivity further. In contrast, the combinations of [MRI + RBD] and [MRI + Hyposmia] maintained the sensitivity of individual clinical non-motor markers while enhancing specificity. Notably, [MRI + Hyposmia] outperformed the performance of [MRI] or [Hyposmia] alone, demonstrating the highest BA (BA = 0.900) and MCC (MCC = 0.586) among the evaluated predictors. Combining each imaging marker with both [RBD] and [Hyposmia] did not differ in performance from combining it with [RBD] alone (Table 2).

**TABLE 2:** Performance of Parkinson’s disease conversion predictors.

PD conversion predictor	Sensitivity	Specificity	PPV	NPV	BA	MCC
RBD	0.333	0.825	0.222	0.892	0.579	0.134
Hyposmia	1.000*	0.575	0.261	1.000*	0.788	0.387
RBD + Hyposmia	0.333	0.850	0.250	0.895	0.592	0.163
MRI	1.000*	0.625	0.286	1.000*	0.813	0.423
MRI + RBD	0.333	0.950*	0.500*	0.905	0.642	0.339
MRI + Hyposmia	1.000*	0.800	0.429	1.000*	0.900*	0.586*
MRI + RBD + Hyposmia	0.333	0.950*	0.500*	0.905	0.642	0.339
DaT	0.833	0.850	0.455	0.971	0.842	0.540
DaT + RBD	0.167	0.925	0.250	0.881	0.546	0.110
DaT + Hyposmia	0.833	0.850	0.455	0.971	0.842	0.540
DaT + RBD + Hyposmia	0.167	0.925	0.250	0.881	0.546	0.110

BA, balanced accuracy; DaT, dopamine transporter imaging; MCC, Matthews correlation coefficient; NPV, negative predictive value; PD, Parkinson’s disease; PPV, positive predictive value; RBD, rapid eye movement sleep behavior disorder. *The highest performance values for each performance metric.

## 4 Discussion

This study aimed to enhance predictive capability for conversion to PD during the prodromal phase by introducing an MRI marker derived from routine MRI sequences and combining it with clinical non-motor markers such as RBD and olfactory dysfunction. The proposed MRI marker facilitated the assessment of whether individuals with prodromal symptoms exhibited brain structural patterns more similar to those of PD individuals than to those of healthy individuals. In predicting conversion to PD within 4 years, the MRI marker demonstrated higher sensitivity but lower specificity compared to established imaging approaches. Notably, when combined with olfactory dysfunction, the MRI marker maintained perfect sensitivity while substantially improving specificity, yielding the most effective overall performance (BA = 0.900 and MCC = 0.586). These findings suggest that the multimodal combination of clinical non-motor and imaging markers, particularly the proposed MRI marker with olfactory dysfunction, could be a promising approach to enhancing predictive performance for conversion to PD in individuals with prodromal symptoms.

The MRI marker demonstrates that brain structural patterns encompassing both GM morphometry and WM integrity can subcategorize the intermediate prodromal stage between the healthy and PD states into the close-to-healthy and close-to-PD states, with the consistent and robust contributions of brain regions to the differentiation between the healthy and PD states, as well as between the close-to-healthy and close-to-PD states. The relatively greater impacts of WM integrity compared to GM morphometry could be associated with the precedence of WM integrity changes over cortical morphological alterations in PD progression (Park et al., 2022). The brain structural patterns predictive of the close-to-PD or PD state, observed as GM atrophy and WM disintegrity primarily in anterior brain regions, may indicate the initial stages of propagating brain structural changes throughout the brain (Potgieser et al., 2014), while concurrent opposing brain structural changes observed in other regions might represent compensatory mechanisms occurring at similar early stages of disease progression (Mole et al., 2016; Sawczak et al., 2019).

The proposed MRI marker exhibited distinct characteristics compared to the DaT scan marker, offering complementary rather than redundant information for PD conversion prediction. While DaT scans assess nigrostriatal dopaminergic integrity with balanced sensitivity (83%) and specificity (85%), our MRI marker achieved higher sensitivity (100%) but lower specificity (63%) when used individually. Importantly, the MRI approach captures structural brain changes that may precede the dopaminergic dysfunction detectable by DaT scans, potentially identifying individuals at earlier stages before substantial nigral cell loss becomes apparent. From a practical standpoint, structural MRI offers significant advantages including wider accessibility, non-invasive acquisition, and lower cost compared to DaT scans, making it particularly valuable in clinical settings where nuclear imaging is not readily available. When combined with clinical markers, the DaT scan marker did not enhance predictive performance, whereas the MRI marker, particularly with olfactory dysfunction, maintained perfect sensitivity while substantially improving specificity to 80%, resulting in superior overall performance (BA = 90%). This suggests that structural MRI may provide complementary neuroanatomical information that, when combined with clinical assessments, offers additional value beyond what is captured by functional dopaminergic imaging alone.

The absence of significant clinical differences between close-to-healthy and close-to-PD states deserves careful interpretation. This lack of significant differences likely reflects that brain structural patterns may capture PD-related alterations that extend beyond those associated with measurable clinical signs and symptoms, potentially preceding the emergence of overt clinical manifestations. Additionally, the prodromal stage represents a continuum where structural changes may be more sensitive than current clinical assessments in detecting early pathological processes, and our sample size may have limited power to detect subtle clinical differences between these intermediate groups. Recent studies have shown that neuroimaging markers can detect pathological alterations that precede clinically measurable symptoms (Yang et al., 2021; Pimer et al., 2023), supporting the concept that structural brain changes represent an intermediate phenotype between normal aging and overt clinical manifestations. This finding actually strengthens the argument for structural imaging as a complementary biomarker, suggesting it captures pathophysiological processes that may not yet be reflected in current clinical scales designed for manifest disease stages. Our results extend this concept by demonstrating that multimodal approaches combining structural imaging with clinical markers can enhance predictive performance compared to individual modalities, as shown in recent PD prediction studies (Makarious et al., 2022; Zhu et al., 2024). The lack of clinical discrimination between our MRI-defined subgroups therefore supports the potential value of structural imaging in identifying individuals at risk before conventional clinical assessments can reliably differentiate risk levels.

Our findings have important clinical implications for prodromal PD management. This approach offers several concrete clinical applications: first, stratification of high-risk individuals for enrollment in clinical trials of disease-modifying treatments, enabling more efficient trial design and potentially accelerating therapeutic development; second, identification of prodromal individuals who may benefit from early monitoring and intervention strategies; and third, development of personalized risk profiles to guide clinical decision-making regarding timing of follow-up assessments and interventions. Given the accessibility and cost-effectiveness of structural MRI compared to specialized nuclear imaging, this multimodal approach presents a scalable strategy for implementation in diverse clinical settings, particularly until more accessible biomarkers such as blood-based assays are developed for PD, as has been achieved in Alzheimer’s disease.

Several important limitations warrant acknowledgment. First, our sample size was constrained by stringent inclusion criteria requiring both structural and diffusion-weighted MRI scans, resulting in a relatively small cohort with only six PD conversions over 4 years. This limitation, while reducing statistical power, reflects the practical challenges of longitudinal prodromal studies and may have contributed to potential overfitting concerns. Second, the 4-years follow-up period may not capture all incident cases due to variable lead times from marker manifestation to PD conversion, particularly affecting RBD assessment which typically has longer lead times (Postuma and Berg, 2016). Third, while we employed uniform preprocessing pipelines and these standardization procedures help reduce variability across sites, we acknowledge that they cannot entirely eliminate all sources of technical variation between different imaging systems and acquisition protocols. Fourth, our focus on structural MRI and clinical non-motor markers excluded other potentially valuable biomarkers such as motor abnormalities with shorter lead times (Postuma et al., 2012b), functional brain alterations (Heinzel et al., 2019), and emerging biomarkers like nigrosomal iron content (Pavese and Tai, 2018). Future studies should address these limitations through larger multicenter collaborations with extended follow-up periods, standardized acquisition protocols, and incorporation of additional biomarker modalities to validate and extend our exploratory findings.

## 5 Conclusion

The findings underscore the potential of multimodal markers in improving the prediction of conversion to PD in individuals with prodromal symptoms. The combination of the MRI-based brain structural marker and olfactory dysfunction demonstrated enhanced overall performance by leveraging the complementary predictive abilities of individual markers, achieving 90% balanced accuracy in identifying future PD converters. A multifaceted predictive framework integrating the complementary abilities of clinical and imaging markers represents a promising avenue for facilitating early identification and management of individuals at risk for PD development.

## Data Availability

Publicly available datasets were analyzed in this study. This data can be found here: Parkinson’s Progression Markers Initiative.

## Footnotes

1
https://www.ppmi-info.org/

2
https://mcin.ca/technology/civet/

3
http://fsl.fmrib.ox.ac.uk/fsl/

4
https://gitlab.com/chang-hyun.park/pd-conversion-prediciton
