# Supplementary material for: Brain structural MRI marker for predicting conversion to Parkinson’s disease in individuals with prodromal symptoms
Source: Front Aging Neurosci. 2025 Jul 16;17:1579326. doi: 10.3389/fnagi.2025.1579326 (PMC12307297; doi:10.3389/fnagi.2025.1579326)
Supplement: Supplementary file 1 [file Data_Sheet_1.pdf]

Supplementary Material for:

Brain Structural MRI Marker for Predicting Conversion to Parkinson's Disease in Individuals with Prodromal Symptoms

Table of Contents:

1. Supplementary Methods
   1.1 Appendix S1. Study sample
   1.2 Appendix S2. Extraction of brain structural features
   1.3 Appendix S3. Development and evaluation of an MRI-based classifier
   1.4 Appendix S4. Confounder-adjusted permutation test
   1.5 Appendix S5. SHAP analysis
   1.6 Appendix S6. Assessment of known PD conversion predictors
   1.7 Appendix S7. Performance assessment of PD conversion predictors

2. Supplementary Results
   2.1 Appendix S8. PD class probabilities of prodromal individuals
   2.2 Appendix S9. Clinical characteristics of prodromal individuals
   2.3 Appendix S10. Brain structural contributions to the division of prodromal individuals

3. Supplementary Tables
   Table S1: Acquisition parameters for MRI data
   Table S2: Confusion matrices given by Parkinson's disease conversion predictors

4. Supplementary Figures
   Figure S1: Comparison of Parkinson's disease class probabilities
   Figure S2: Brain maps depicting the magnitude of Shapley additive explanations

Supplementary Methods

Appendix S1. Study sample

Prodromal individuals

As determined by the Parkinson's Progression Markers Initiative (PPMI), participants eligible for inclusion in the prodromal cohort were 60 years of age or older, had no clinical diagnoses of Parkinsons's disease (PD), other forms of parkinsonism, or dementia, and had specific risk factors or clinical markers associated with PD. In addition, inclusion of prodromal individuals in this study was specifically contingent upon the following criteria: (i) the presence of screening outcomes for rapid eye movement sleep behavior disorder (RBD), olfactory dysfunction, and nigrostriatal dopaminergic neurodegeneration, (ii) the presence of diagnostic outcomes from follow-ups for more than four years, and (iii) availability of structural and diffusion-weighted MRI scans at baseline. As illustrated in the flowchart in Figure 1, individuals were excluded from the study based on predefined criteria, which encompassed cases with incomplete or anomalous data and, particularly for MRI scans, instances where data processing errors occurred.

Healthy and PD individuals

Both healthy individuals and those diagnosed with PD were included from the PPMI dataset to serve as training and test sets and from the local outpatient clinic dataset to serve as an independent test set for developing an MRI-based classifier. The healthy and PD individuals in the training and test sets were selected from their respective cohorts, as defined by the up-to-date criteria of the PPMI, contingent upon the availability of both structural and diffusion-weighted MRI scans at baseline. For the independent test set, the healthy individuals were selected as those without neurological or psychiatric disorders, and the PD individuals were chosen as those with PD onset after 40 years of age, with both groups having accessible structural and diffusion-weighted MRI scans. According to the same criteria applied to the prodromal individuals, cases with incomplete or anomalous data, as well as those where errors occurred during MRI scan processing, were excluded. The final cohort composition was adjusted to maintain, as far as possible, comparable ratios of healthy individuals to those with PD across the training, test, and independent test sets.

Appendix S2. Extraction of brain structural features

Cortical thickness of grey matter (GM) regions was computed using CIVET tools (https://mcin.ca/technology/civet/) through cortical morphometric analysis of structural T1-weighted MRI data. Following reconstruction of hemispheric cortical surfaces, the mean of vertex-wise cortical thickness values was calculated for each of 62 cortical GM regions delineated according to the Desikan–Killiany–Tourville atlas [1]. White matter (WM) integrity, quantified by fractional anisotropy (FA), was computed using FSL tools (http://fsl.fmrib.ox.ac.uk/fsl/) through diffusion tensor analysis of diffusion-weighted MRI data. After projecting FA values onto an alignment-invariant tract representation, the mean of voxel-wise FA values was calculated for each of 48 WM regions parcellated according to the ICBM DTI-81 atlas [2].

Appendix S3. Development and evaluation of an MRI-based classifier

The dataset comprising the healthy (n = 75) and PD (n = 132) individuals was partitioned into the training (80% of the individuals; 60 healthy and 106 PD individuals) and test (remaining 20% of the individuals; 15 healthy and 26 PD individuals) sets, maintaining the class distribution ratio. For the training set, a random forest model was implemented using the scikit-learn package (https://scikit-learn.org/; version 1.3.2). Through five-fold cross-validation, we optimized several hyperparameters: we used 200 trees in the forest (n_estimators=200), with no maximum depth limit for each tree (max_depth=None). For node splitting, we required a minimum of 2 samples (min_samples_split=2) and set the minimum samples at leaf nodes to 1 (min_samples_leaf=1). The number of features considered for the best split was set to the square root of the total features (max_features='sqrt'). We did not employ bootstrap sampling when building trees (bootstrap=False) and used no special class weights (class_weight=None). The function used to measure split quality was the Gini impurity (criterion='gini'). These hyperparameters were selected based on their performance in correctly classifying healthy and PD individuals in the inner cross-validation folds, with optimization prioritizing balanced performance across sensitivity and specificity. The resultant model was then applied to the test set to predict the class membership of individuals excluded from training.

Additionally, for external validation, the model was employed to generate predictions on the separate, independent test set (83 healthy and 130 PD individuals).

Appendix S4. Confounder-adjusted permutation test

To compare scores on clinical assessments between groups, a confounder-adjusted permutation test was conducted according to the following process: (i) confounders including age and sex were adjusted for by regressing the outcome variable against them and retaining the residuals, (ii) the null distribution of a nonparametric test statistic (from the Kruskal–Wallis test for the comparison between all groups or from the Mann–Whitney U test for post hoc pair-wise comparisons) under the assumption of no group effect was generated by permuting the group labels 1,000 times, each time recalculating the test statistic on the permuted groups, and (iii) a P value was computed by comparing the observed statistic to the distribution of permuted statistics.

Appendix S5. SHAP analysis

Shapley additive explanations (SHAP), specifically TreeSHAP [3] proposed for tree-based machine learning models, was employed to elucidate the significance of each brain structural feature on the prediction of the classifier. By implementing SHAP as in the respective Python package (https://github.com/shap/shap), the classifier's prediction was deconstructed into a sum of contributions from the features, providing SHAP values that reflected how informative the features were in relation to the division of the prodromal individuals into the close-to-healthy and close-to-PD states, as well as the distinction between healthy and PD brains.

Appendix S6. Assessment of known PD conversion predictors

Screening for olfactory dysfunction

In the PPMI dataset, the University of Pennsylvania smell identification test (UPSIT) was conducted to evaluate olfactory function. The test was scored based on the number of correct identifications out of 40 items. According to the age- and sex-adjusted criteria, scores of 18 or lower for both males and females were categorized as anosmia, while scores between 18 and 33 for males and between 18 and

34 for females were categorized as hyposmia. Olfactory dysfunction was operationally defined as the presence of either hyposmia or anosmia, encompassing the spectrum of reduced olfactory function, whereby we defined individuals with scores of 33 or lower for males and 34 or lower for females as having olfactory dysfunction.

Screening for RBD

In the PPMI dataset, the RBD screening questionnaire (RBDSQ) was conducted to assess the likelihood of RBD. For the questionnaire that consisted of several items that inquire about sleep behavior, responses were scored to indicate the presence or severity of symptoms. Among the given items, if there were missing responses, the RBD score was considered missing. Scores of 5 or higher were categorized as RBD positive (suggestive of probable RBD), while scores below 5 were categorized as RBD negative (suggestive of unlikely RBD). The PPMI protocol excluded individuals with known secondary causes of RBD and allowed investigator discretion to exclude participants based on other medical or psychiatric conditions that could preclude study participation. However, we acknowledge that questionnaire-based assessment may lead to misdiagnosis, as it cannot definitively distinguish idiopathic RBD from secondary forms or other sleep disorders, which represents a limitation in our RBD marker interpretation.

Screening for nigrostriatal dopaminergic neurodegeneration

In the PPMI dataset, dopamine transporter (DaT) scans were performed using single-photon emission computed tomography to assess nigrostriatal dopaminergic degeneration. These scans employed the radioactive tracer Ioflupane (123I), which binds to dopamine transporters in the brain, allowing for the visualization and assessment of dopamine transporter levels. Visual assessments to evaluate the density of dopamine transporters were performed on the images that underwent processing steps such as attenuation correction, filtering, and normalization to enhance quality and consistency. Two independent reviewers initially assessed the scans, and if their interpretations differed, a third reviewer was involved to reach a consensus. Final interpretations were categorized as positive (suggestive of dopaminergic deficit) or negative (suggestive of a normal dopaminergic system) based

on the visual appearance of the striata, particularly the shape and intensity of activity.

Appendix S7. Performance assessment of PD conversion predictors

The performance of each predictor for conversion to PD within a four-year follow-up period was assessed by computing performance metrics, such as sensitivity, specificity, positive predictive value (PPV), and negative predictive value (NPV) defined as follows:

$$\text{Sensitivity} = \frac{\text{TP}}{\text{TP} + \text{FN}},$$

$$\text{Specificity} = \frac{\text{TN}}{\text{TN} + \text{FP}},$$

$$\text{PPV} = \frac{\text{TP}}{\text{TP} + \text{FP}}, \text{ and}$$

$$\text{NPV} = \frac{\text{TN}}{\text{TN} + \text{FN}}$$

where TP, TN, FP, and FN are true positives, true negatives, false positives, and false negatives, respectively. Furthermore, balanced accuracy (BA) and Matthews correlation coefficient (MCC) were computed as comprehensive performance metrics that effectively evaluate the quality of binary classifications by assessing outcomes for each class proportionally as follows:

$$\text{BA} = \frac{\text{TP}/(\text{TP} + \text{FN}) + \text{TN}/(\text{TN} + \text{FP})}{2} = \frac{\text{Sensitivity} + \text{Specificity}}{2} \text{ and}$$

$$\text{MCC} = \frac{(\text{TP} \times \text{TN}) - (\text{FP} \times \text{FN})}{\sqrt{(\text{TP} + \text{FP})(\text{TP} + \text{FN})(\text{TN} + \text{FP})(\text{TN} + \text{FN})}}.$$

While BA considers the balance between TP and TN, providing a metric ranging from 0 to 1, where 0.5 is no better than random guessing, MCC takes into account all four quadrants of the confusion matrix, including TP, TN, FP, and FN, providing a metric ranging from -1 to +1, where 0 is no better than random guessing.

Supplementary Results

Appendix S8. PD class probabilities of prodromal individuals

The mean ± standard deviation of PD class probabilities was 0.499 ± 0.141 for all the prodromal individuals; it was significantly high for those in the close-to-PD state (0.616±0.087) than those in the close-to-healthy state (0.392±0.084) (close-to-healthy vs. close-to-PD: $t = -8.894$, $P < .001$) (Figure S1). Whereas PD class probabilities were not significantly different between the prodromal individuals in the close-to-healthy state and the healthy individuals (0.338±0.163), the prodromal individuals in the close-to-PD state exhibited lower PD class probabilities than the PD individuals (0.797±0.138) (close-to-PD vs. PD: $t = -5.326$, $P < .001$).

Appendix S9. Clinical characteristics of prodromal individuals

When scores on clinical assessments of the prodromal individuals according to their designated states were compared with those of the healthy and PD individuals (Figure 2), significant differences in mean scores were shown for the Hoehn and Yahr staging scale (HYSS) ($X^2 = 184.688$, $P < .001$), Movement Disorder Society-sponsored revision of the unified PD rating scale part III (MDS-UPDRS III) ($X^2 = 183.853$, $P < .001$), RBDSQ ($X^2 = 12.767$, $P = .005$), and UPSIT ($X^2 = 76.257$, $P < .001$). In post hoc pair-wise comparisons, both states of the prodromal individuals exhibited lower HYSS scores than the PD individuals (close-to-healthy vs. PD: $z = -7.846$, $P < .001$; close-to-PD vs. PD: $z = -6.959$, $P < .001$), while they presented with higher RBDSQ scores than the healthy individuals (close-to-healthy vs. healthy: $z = 2.420$, $P = .008$; close-to-PD vs. healthy: $z = 2.570$, $P = .008$). For the MDS-UPDRS III, both states of the prodromal individuals displayed lower scores than the PD individuals (close-to-healthy vs. PD: $z = -7.697$, $P < 0.001$; close-to-PD vs. PD: $z = -6.800$, $P < .001$) and higher scores than the healthy individuals (close-to-healthy vs. healthy: $z = 2.642$, $P = .004$; close-to-PD vs. healthy: $z = -2.982$, $P = .002$). For the UPSIT, among the prodromal individuals, those in the close-to-healthy state exhibited higher scores than the PD individuals (close-to-healthy vs. PD: $z = 3.082$, $P < .001$), while those in the close-to-PD state showed lower scores than the healthy individuals (close-to-PD vs. healthy: $z = -3.390$, $P < .001$).

Appendix S10. Brain structural contributions to the division of prodromal individuals

The directionality of changes in the brain structural features towards the close-to-PD or PD state broadly indicated the occurrence of cortical thinning and WM disintegrity primarily in anterior brain regions, such as the orbitofrontal and anterior cingulate cortices, genu of the corpus callosum, uncinate fasciculus, and anterior limb of internal capsule, while opposing directional changes were also observed in other brain regions, such as the inferior parietal and posterior cingulate cortices, cuneus, and medial lemniscus.

Supplementary Tables

Table S1. Acquisition parameters for structural and diffusion-weighted MRI data.

Data role	Data source	Participants	Structural MRI (3D T1WI)			Diffusion-weighted MRI (DTI)			
			**Voxel size (mm)**	**Slice thickness (mm)**	**Imaging plane**	**Voxel size (mm)**	**Slice thickness (mm)**	**Diffusion gradient directions**	**Imaging plane**
Training, test, and target sets	PPMI dataset	Healthy, prodromal, and PD	1.0 × 1.0	1.0-1.2	Sagittal	1.98-2.0 × 1.98-2.0	2.0	64	Axial
Independent test set	Local outpatient clinic dataset	Healthy, PD	1.0 × 1.0	1.0	Coronal	2.0 × 2.0	2.0	32	Axial

Abbreviations: DTI = diffusion tensor imaging, PD = Parkinson's disease, PPMI = Parkinson's Progression Markers Initiative, T1WI = T1-weighted imaging.

Table S2. Confusion matrices given by Parkinson's disease conversion predictors

PD conversion predictor	TP	TN	FP	FN	Odds ratio	*P* value*
RBD	2	33	7	4	2.357	.33
Hyposmia	6	23	17	0	Infinity	.01
RBD + Hyposmia	2	34	6	4	2.833	.28
MRI	6	25	15	0	Infinity	.01
MRI + RBD	2	38	2	4	9.500	.08
MRI + Hyposmia	6	32	8	0	Infinity	< .001
MRI + RBD + Hyposmia	2	38	2	4	9.500	.08
DaT	5	34	6	1	28.333	.002
DaT + RBD	1	37	3	5	2.467	.44
DaT + Hyposmia	5	34	6	1	28.333	.002
DaT + RBD + Hyposmia	1	37	3	5	2.467	.44

Abbreviations: FN = false negative, FP = false positive, PD = Parkinson's disease, TN = true negative, TP = true positive.

* *P* values represent the statistical significance of the association between predicted and actual PD conversion outcomes.

Supplementary Figures

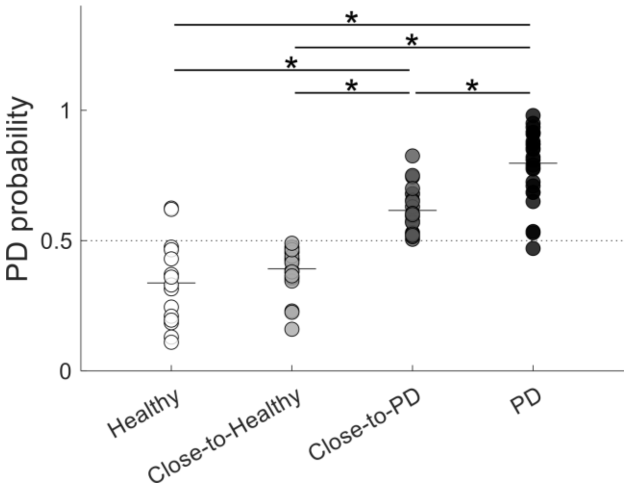

Figure S1. Comparison of Parkinson's disease (PD) class probabilities. PD class probabilities of individuals with prodromal symptoms of PD in the close-to-healthy or close-to-PD state are compared to healthy individuals and those with PD. Each dot in the scatter plot represents an individual's probability, while horizontal lines within each group depict the mean probability across the individuals. Statistically significant differences in post-hoc pair-wise comparisons are indicated by asterisks above the compared groups.

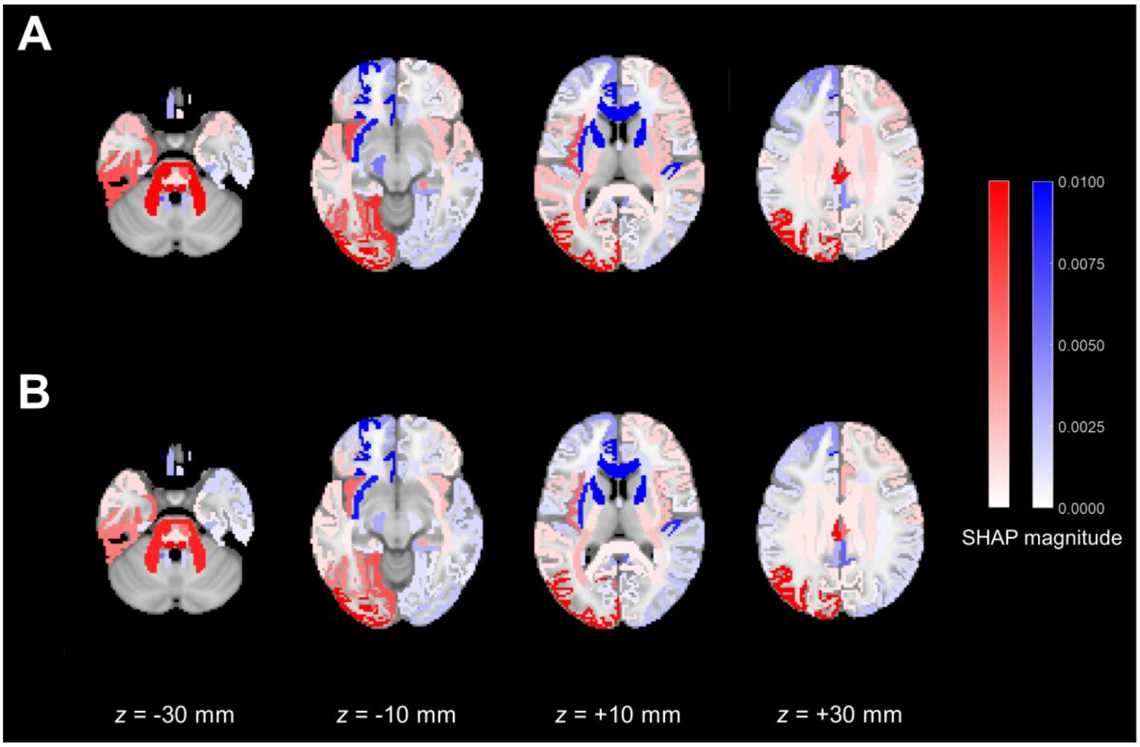

Figure S2. Brain maps depicting the magnitude of Shapley additive explanations (SHAP). In brain slices, SHAP magnitude, calculated as the mean of absolute SHAP values across samples, reflects the relevance of various brain regions to the classifier's prediction for (A) dividing individuals with prodromal symptoms of Parkinson's disease (PD) into the close-to-healthy and close-to-PD states and for (B) distinguishing between healthy and PD brains. Levels of SHAP magnitude are visualized with a color scale where red indicates a positive correlation (higher feature values push prediction towards the close-to-PD or PD state versus the close-to-healthy or healthy state) and blue indicates a negative correlation (lower feature values push prediction towards the close-to-PD or PD state versus the close-to-healthy or healthy state) between feature values and SHAP values in the respective brain regions. Each panel progresses from inferior to superior axial slices as indicated by *z*-coordinates in millimeters (mm), with the left hemisphere of the brain presented on the left and the right hemisphere on the right.

Supplementary References

1 Klein A, Tourville J (2012) 101 labeled brain images and a consistent human cortical labeling protocol. Front Neurosci 6:171

2 Mori S, Oishi K, Jiang H et al (2008) Stereotaxic white matter atlas based on diffusion tensor imaging in an ICBM template. Neuroimage 40:570-582

3 Lundberg SM, Erion GG, Lee S-I (2018) Consistent individualized feature attribution for tree ensembles. arXiv preprint arXiv:180203888
